# Natural-focal diseases: mapping experience in Russia

**DOI:** 10.1186/1476-072X-13-21

**Published:** 2014-06-14

**Authors:** Svetlana M Malkhazova, Varvara A Mironova, Tatiana V Kotova, Natalia V Shartova, Dmitry S Orlov

**Affiliations:** 1 Department of Biogeography, Faculty of Geography, Lomonosov Moscow State University, GSP-1, Leninskie Gory, Moscow 119991, Russian Federation; 2 Scientific Laboratory of Complex Mapping, Faculty of Geography, Lomonosov Moscow State University, GSP-1, Leninskie Gory, Moscow 119991, Russian Federation; 3 Department of Landscape Geochemistry and Soil Geography, Faculty of Geography, Lomonosov Moscow State University, GSP-1, Leninskie Gory, Moscow 119991, Russian Federation

**Keywords:** Russia, Distribution and diversity of natural-focal diseases, Spatiotemporal dynamics, GIS, Medico-geographical atlas

## Abstract

**Background:**

Natural-focal diseases constitute a serious hazard for human health. Agents and vectors of such diseases belong to natural landscapes. The aim of this study is to identify the diversity and geography of natural-focal diseases in Russia and to develop cartographic approaches for their mapping, including mathematical-cartographical modeling. Russian medico-geographical mapping of natural-focal diseases is highly developed regionally and locally but extremely limited at the national level. To solve this problem, a scientific team of the Faculty of Geography at Lomonosov Moscow State University has developed and implemented a project of a medico-geographical Atlas of Russia “Natural-Focal Diseases”.

**Methods:**

The mapping is based on medical statistics data. The Atlas contains a series of maps on disease incidence, long-term dynamics of disease morbidity, etc. In addition, other materials available to the authors were used: mapping of the natural environment, field data, archival materials, analyzed satellite images, etc. The maps are processed using ArcGIS (ESRI) software application. Different methods of rendering of mapped phenomena are used (geographical ranges, diagrams, choropleth maps etc.).

**Results:**

A series of analytical, integrated, and synthetic maps shows disease incidence in the population at both the national and regional levels for the last 15 years. Maps of the mean annual morbidity of certain infections and maps of morbidity dynamics and nosological profiles allow for a detailed analysis of the situation for each of 83 administrative units of the Russian Federation. The degree of epidemic hazard in Russia by natural-focal diseases is reflected in a synthetic medico-geographical map that shows the degree of epidemic risks due to such diseases in Russia and allows one to estimate the risk of disease manifestation in a given region.

**Conclusions:**

This is the first attempt at aggregation and public presentation of diverse and multifaceted information about natural-focal diseases in Russia. Taken in entirety, the maps that have been prepared for the Atlas will enable researchers to evaluate the stability of epidemic manifestation of individual diseases and the susceptibility of a given territory to disease transmission. The results can be used for sanitary monitoring and disease prevention.

## Introduction

Natural-focal disease prevention is one of the most important problems of public health. The agents and vectors of these diseases are part of natural landscapes and the spread of these diseases, which may be a serious hazard for the population, is determined by natural factors. Therefore, medical geography has an important task: evaluating the risk of epidemic hazard of natural ecosystems and providing public health authorities with recommendations necessary to prevent disease outbreaks and conduct epidemiological surveillance.

In accordance with a theory of focality (or nidality) of disease proposed by Russian academician Eugene Pavlovsky in 1939, some pathogens are associated with specific landscapes [1]. Natural focus or nidus is the central, crucial concept of Pavlovsky’s theory. According to a recent conception resulting from 75 years of scientific investigations in Russia and abroad, the term “natural focus of an infectious disease” refers to any natural ecosystem in which a pathogen population is an essential component [2]. The determinant feature of natural-focal disease is that the pathogen of such a disease circulates in the nature independently of human presence. As a rule, the humans became infected when they get into the focus and contact the infectious vector or, in some cases, the reservoir host [3].

In Pavlovsky’s original theory, based on tick-borne pathogens in Russia, the focus of infection contains three critical elements: vectors, vertebrate hosts, and susceptible recipient hosts such as humans or animals. Nowadays, the natural focality has been proved also for non-vector-borne zoonoses such as hemorrhagic fever with renal syndrome, leptospirosis, etc. Finally, natural focality for a large group of sapronotic infections, whose agents live in soil or aquatic ecosystems, has also been substantiated. For some vector-borne anthroponoses the concept of a focus (nidus) may be implemented as well. So, the phenomenon of natural focality is widespread, and there are a lot of natural-focal diseases with different types of transmission.

In the past two decades views on the diversity, spread, and epidemic significance of infections with natural focality have changed substantially all over the world. Some new pathogens have been discovered and periodic epizootic and epidemic manifestations of natural foci have become a matter of great concern. Morbidity due to some natural-focal diseases such as tick-borne encephalitis and ixodid tick-borne borreliosis (the Palaearctic analog of Lyme disease that is widespread in the North America), as well as some helminthoses with natural focality, such as opisthorchiasis, remains high in the Russian Federation.

Therefore, we deal with a broad range of natural-focal diseases that may harm the population and visitors of different regions of Russia.

In recent decades, increasing human activities (e.g., intensive suburban construction around big cities, expansion and growth of recreational pressure) have led to a significant increase in contact between the population and the natural foci and in favorable epidemiological conditions for the spread of natural-focal diseases [4,5].

This paper examines the experience of medico-geographical research in the area of natural-focal diseases in Russia and presents the opportunities given by the mapping method for the assessment and monitoring of the epidemiological situation with respect to a number of dangerous diseases.

Despite the increased attention to this issue in the past decade [6-13], many research questions pertaining to natural-focal diseases remain unanswered. Development of the principles and methods of synthesizing medico-geographical information and obtaining new knowledge about the spatial distribution patterns of natural-focal diseases using new approaches remain primary research interests.

One important aspect of such studies is atlas mapping, which combines general scientific methods (integrated, historical, etc.) and specific (statistical, landscape, medico-geographical, etc.) approaches with geographical information technologies [14-18].

Currently, a considerable amount of different medico-geographical atlases have been published around the world. Some of them exist in a print version, others are available online, and they cover a vast range of topics – from general characteristics of the current distribution and determinants of major human infectious diseases [7,19] to regional atlases [20,21] or atlases that focus on particular diseases (malaria, plague, helminth diseases etc.) [6,22,23]. These publications aim to disseminate up-to-date information on some of the most important diseases all over the world. Some atlases reflect the epidemiological situation in context of environmental factors (e.g., WHO/WMO “The Atlas of health and climate” [24], which is a product of collaboration between meteorological and public health communities, provides sound scientific information on the connections between weather, climate and major health challenges).

The experience of Soviet and Russian medico-geographical mapping is also rather extensive. The scientific and methodological basis of medico-geographical mapping that uses the landscape approach, methods of mathematical statistics, multivariable analysis, interconnected mapping studies, and synthesis of the information is well developed [25-27]. However, the scientific-methodological and practical experience of the national medico-geographical mapping, in particular, mapping of natural-focal diseases is significant in the field of regional and local mapping only but is extremely limited in the mapping at the national level. Differences in quality and incompleteness of initial information and the use of different methodological mapping approaches make it difficult to obtain a complete picture of the distribution of natural-focal diseases within the territory of the Russian Federation. A cartographic summary showing the geography of natural-focal diseases for Russia as a whole is still lacking.

The first and the only general map of natural-focal diseases for the territory of the former Soviet Union at a 1:25,000,000 scale was published in 1964 [25]. That map was very informative for its time and eminently reflected the existing situation, although the geography of natural-focal diseases was under-explored, and some natural-focal infections hadn’t been discovered. At present, accounting for availability of information about new diseases and significant changes of the environment that occurred during the last fifty years, this map doesn’t reflect the actual situation. Thus, it is currently necessary to conduct a new cartographic study on natural-focal diseases in Russia. Without development of maps that reflect the situation for the entire Russian territory, it is impossible to provide informational support for sound monitoring of the epidemiological situation in the country and its regions.

The results of such a study can be presented as an atlas information system both in print and in GIS. For such a vast country as Russia, which contains a wide range of natural zones, from tundras in the north to deserts in the south, this sort of a multilevel system may provide an idea of the current situation and serve as a useful model for the development of similar geoinformation systems for other areas.

Considering the importance of the problem, a team of researchers at the Faculty of Geography of Lomonosov Moscow State University (Russia) has developed a medico-geographical Atlas of Russia “Natural-Focal Diseases” [28-30].

### The conceptual design and structure of the Atlas

The Atlas was developed in accordance with the following principles of medico-geographical research:

• each natural ecosystem has its own specific “set” of natural preconditions of human diseases, i.e., each landscape has its own typical parasitogenic systems formed in specific environmental conditions;

• the incidence natural-focal diseases are closely connected with the existing natural, socioeconomic, and demographic characteristics of the territory;

• identification of causal relationships between the nosological situation (potential risk) of the environment and public health and their appropriate representation is possible only with a systematic approach;

• natural-focal diseases and geographical preconditions of diseases are dynamic structures governed by the laws of geosystem evolutionary development and the characteristics of economic development of territories; adequacy of their mapping can be achieved only when considering dynamic changes of the studied phenomena;

• the general character of cartographic data assumes the use of extrapolation and analogy methods in analysis and synthesis of spatial information at the national level.

The Atlas structure is a combination of cartographic, textual, and graphic information, photographs, and analytical data. The spatial distribution of natural-focal diseases is considered at different hierarchic levels. The Atlas has seven thematic blocks and more than 100 maps.

The *Introductory section* covers the theory of natural-focal infections and invasions as a complex set of interdependent populations of pathogens, animal hosts and arthropod vectors that represent a biological component organically bound with natural territorial complexes.

The *Natural conditions block* comprehensively describes the characteristics and current conditions of the natural environment that influence the formation and functioning of the parasitogenic disease systems (agent–vector–host) in the territory of the Russian Federation.

The *Demographic and socioeconomic conditions block* includes maps on the patterns of the population distribution within the Russian territory and on its demographic indices and is intended to identify specific territorial features of the population structure and to treat the population as a component associated with the epidemiological risk of the territory.

The *Natural hosts and vectors of natural-focal diseases block* contains the maps of geographic ranges of animals - potential hosts of infections.

The *Geographic ranges of natural-focal diseases block* contains important maps of the distribution ranges of natural-focal infections and with synthetic maps of medico-geographical assessment of the risk associated with natural-focal diseases in the territory of Russia.

The *Disease morbidity block* contains maps based on the official statistics that can be easily updated as new information becomes available, showing the average long-term incidence of certain natural-focal infections and its multiannual dynamics.

The *Organization of public health system block* reflects the spatial structure of public health service in Russia.

### Data and methods

The background data for the Atlas included Federal State Statistics Service statistics on socioeconomic indicators and the data of the Federal Service on Customers’ Rights Protection and Human Well-Being Surveillance; these sources contain statistical data on disease incidence in the population (for infectious and parasitic diseases). In addition, the Atlas used other materials available to the authors: natural environment mapping; previously compiled maps of the federal and regional atlases; field data and archival material; and the results of satellite image interpretation.

The scope of work included the following steps: 1) preparation, update, and analysis of the database on infectious and parasitic natural-focal diseases; 2) development of a series of maps, including the compilation of analytical (inventory) maps and of integrated and synthetic (assessment) maps; 3) a detailed cartographical study of selected key regions; and 4) medico-geographical analysis based on the compiled maps.

Taking into account the significance of particular diseases and availability of statistical data, 22 nosological units were selected for mapping: anthrax, brucellosis, hemorrhagic fever with renal syndrome (HFRS), legionellosis, leptospirosis, ornithosis, pseudotuberculosis, Q fever, rabies, tetanus, tick-borne borreliosis, tick-borne encephalitis, tick-borne rickettsiosis of North Asia, tularemia, beef tapeworm infection, diphyllobothriasis, echinococcosis, opisthorchiasis, taeniasis, toxocariasis, trichinosis, and trichuriasis.

**Figure 1:**
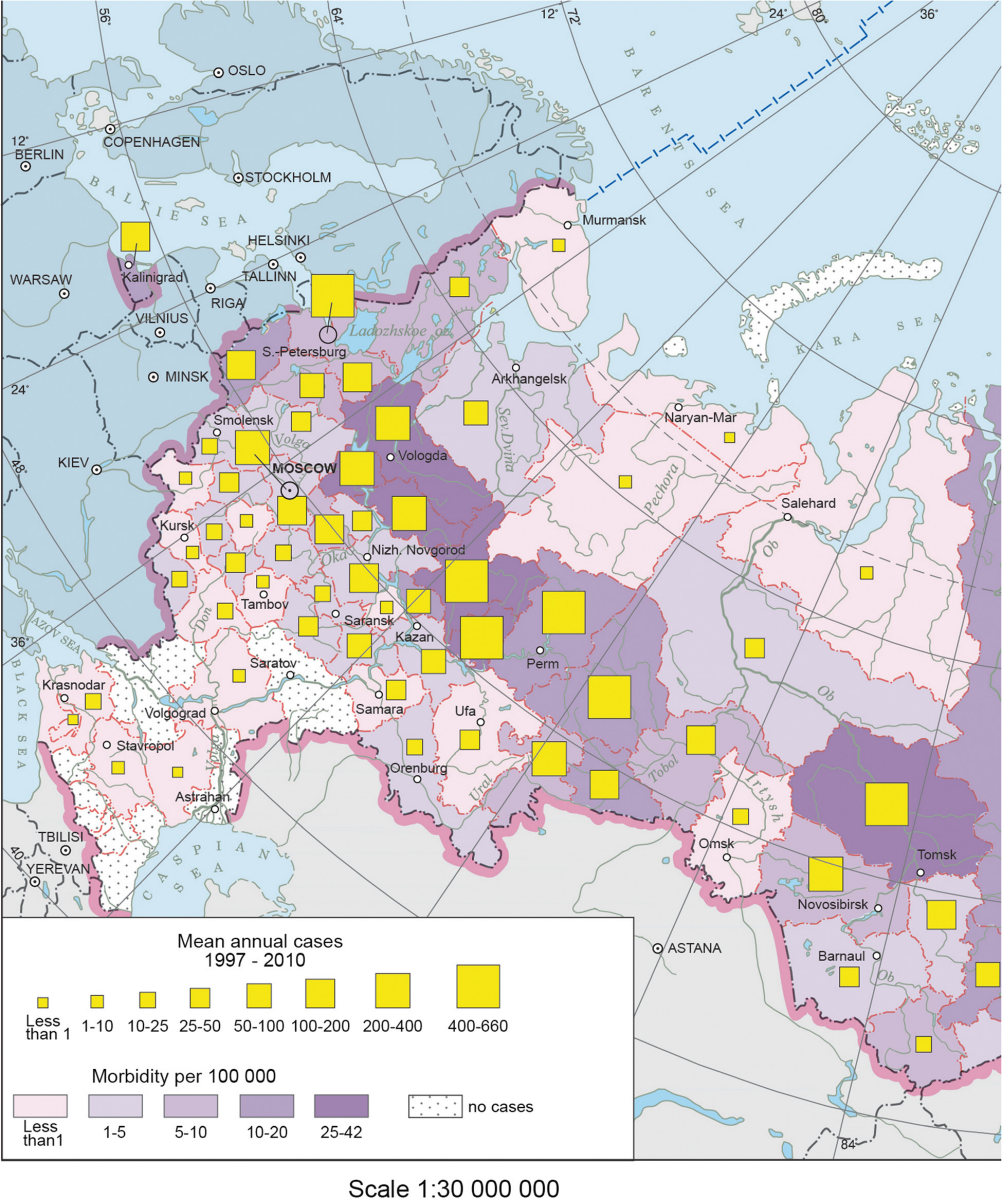
**Morbidity of tick-borne borreliosis (a map fragment of Russian Federation).** The map of the mean annual tick-borne borreliosis morbidity were compiled using the cartogram method and reflect the relative and absolute values for each administrative unit.

Based on statistical data from 1997–2012, a database containing the information for all 83 administrative units of Russia was created. The level of detail of the input data allowed the researcher to use the administrative divisions of the Russian Federation (republics, autonomous districts, territories, districts, and cities of federal significance) as the basic mapping units. The structure of the thematic database was designed to allow for a timely update of the existing tables with the data on new research objects at different administrative-territorial levels. The developed spatial database (including map compilation, rendering, editing, analysis, etc.) utilizes ArcGIS (ESRI) software. The Atlas uses different methods of mapped phenomena rendering: geographical ranges, diagrams (for absolute indices), choropleth maps (for relative indices – number of cases per 100.000 populations), etc.The inventory maps on the long-time average annual morbidity utilize choropleth and diagram methods to represent relative and absolute indices for each administrative unit for a specific period of time. These maps show the level of disease incidence for a given period An example is shown in Figure 1. With their help, one can evaluate the general characteristics of a specific disease in a particular area, identify the most affected regions, and improve the understanding of the natural confinedness of the endemic territories. These maps can also be easily updated with new temporal data. A graded scale was applied to all the inventory maps, the grades being the same for each represented parameter within the analyzed period of time. Almost all maps of the Atlas integrate different representations of several parameters. This technique of data presentation allows for an analysis of the acuity of sanitary-epidemiological situation within the territory under study.

**Figure 2:**
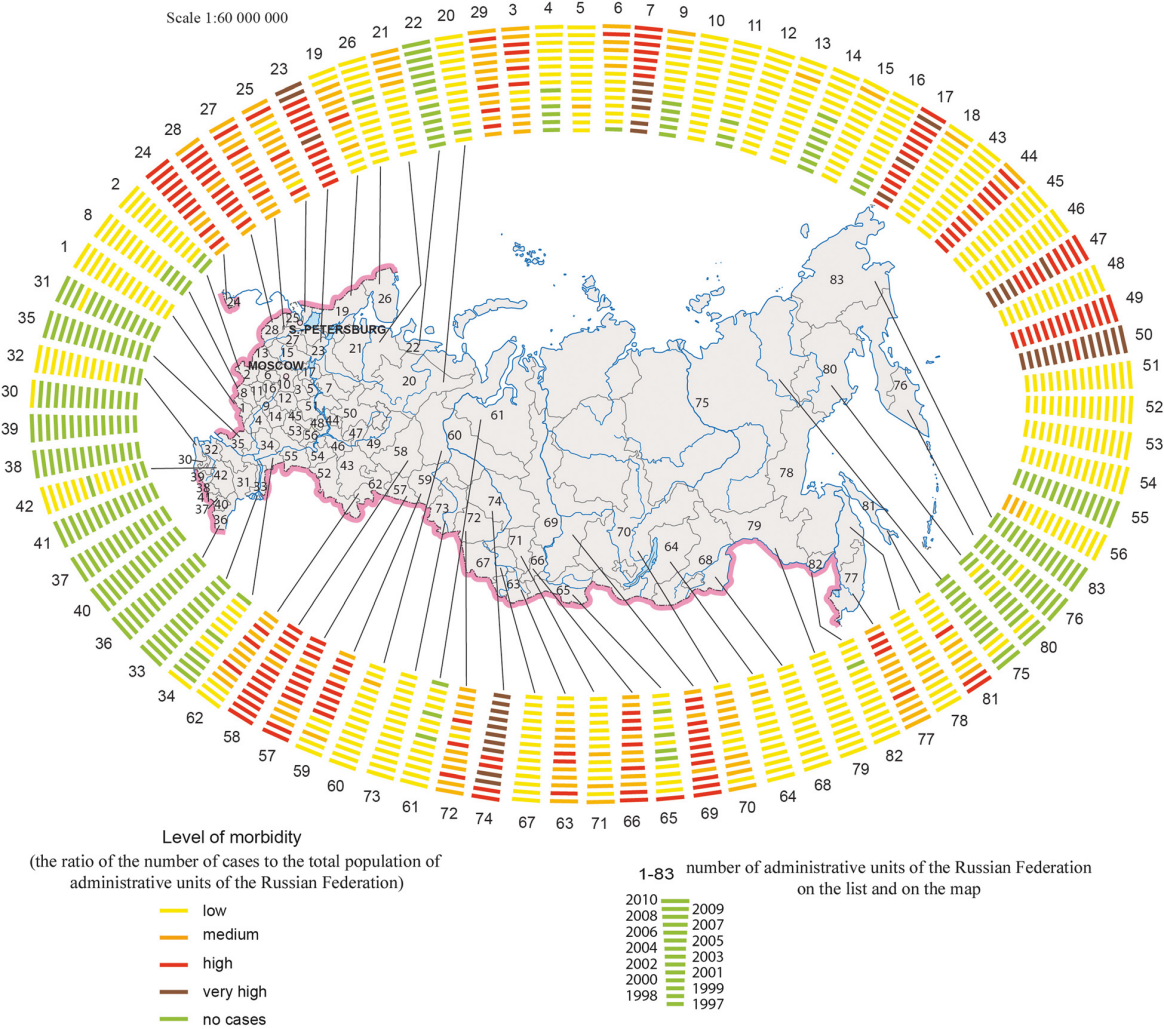
**Dynamics of tick-borne borreliosis morbidity (1997–2010).** The bars attributed to the particular administrative unit (marked by figures from 1 to 83) reflect the dynamics of tick-borne borreliosis morbidity for a time span of 14years. Coloring of bar divisions reflect relative morbidity levels.

**Figure 3:**
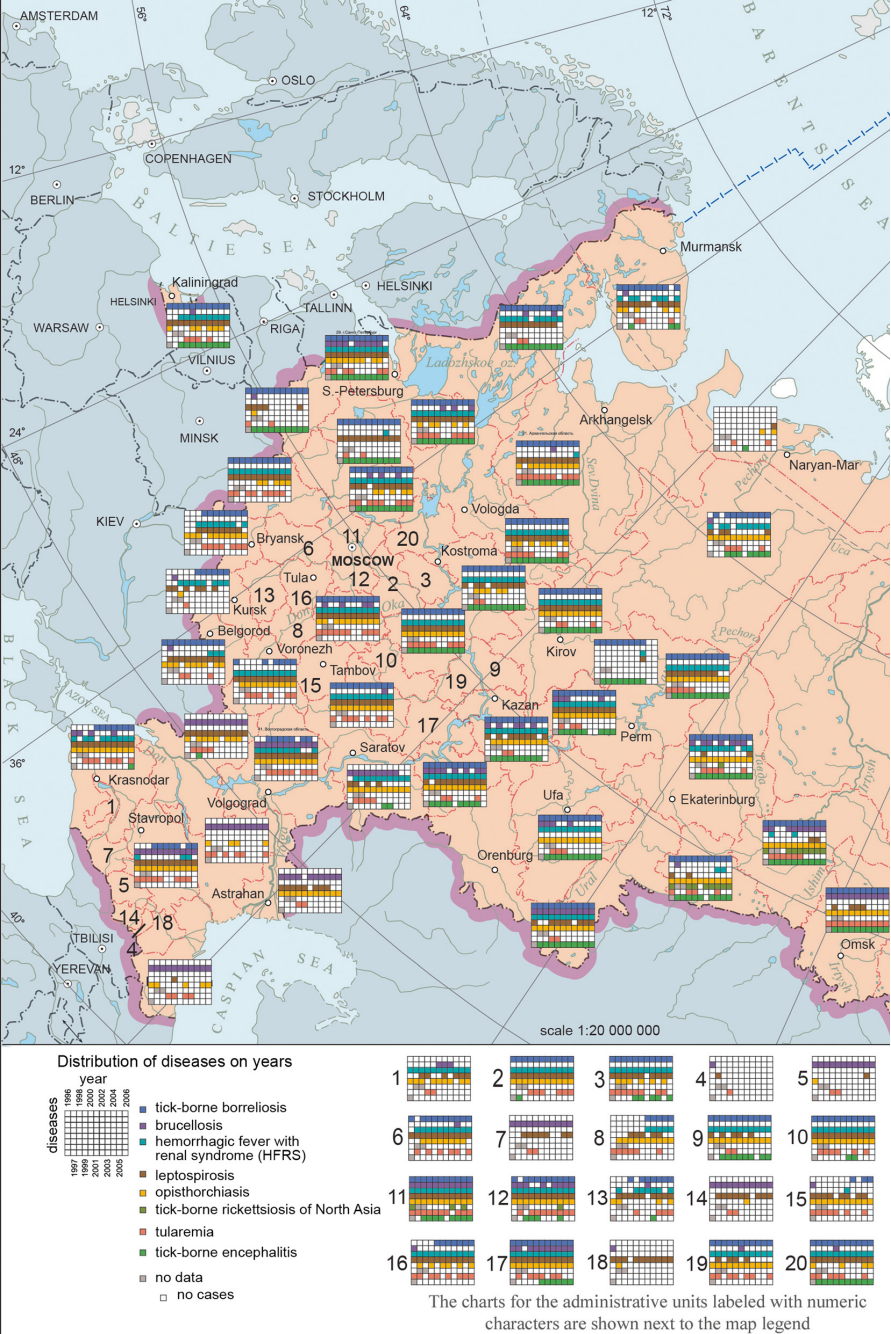
**Nosological profiles of administrative units (a map fragment of Russian Federation).** The map shows the sets of specific natural-focal diseases inherent in particular administrative units of Russia. The data is presented in the form of a matrix showing the presence or absence of a disease in the population for each year for a time span of 11years. The matrices are arranged vertically by nosological units (diseases) and horizontally - by years and they reflect the annual changes in the range of nosoforms in the Russian administrative units.

A typological classification of disease dynamics was conducted based on the analysis of morbidity indicators for 1997–2010. The results of this analysis are reflected in a map of the typological classification of dynamics of population morbidity in the Russian Federation. The map shows different types of taxons obtained from calculated indices; each type has its standard pattern of population morbidity dynamics for a given period (decrease, increase, fluctuations with high or low amplitudes - five taxons in total). The disease dynamics graphics are given in the legend. The map allows for an assessment of the level of stability of epidemiological manifestations of natural foci for the five most important natural-focal diseases of Russia: tick-borne encephalitis, tick-borne borreliosis, hemorrhagic fever with renal syndrome, tick-borne diphyllobothriasis, and opisthorchiasis.A new ring cartogram method was used to show the levels of disease morbidity in different federal administrative units. On the basis of cartographic modeling, all administrative units were divided into 4 estimate groups (low, medium, high and very high) in accordance with an average disease morbidity level from 1997 to 2012. These groups were arranged in the form of a ring cartogram that allows one to assess the role of a certain infection in a given region and identify vulnerable territories. An example is shown in Figure 2.On the basis of the inventory maps, the map “Nosological Profiles” was prepared (Figure 3). The map shows the combinations of specific natural-focal diseases in the administrative divisions, which are presented in the form of a matrix that shows the presence or absence of a disease in the population for each year in a given period. The matrices are arranged vertically and horizontally by nosological units (diseases) and year, respectively. The map provides a composite picture of the spatial-temporary distribution of natural-focal disease in administrative units without an indication of incidence levels.

The Atlas contains medico-geographical maps for key regions related to several diseases, which were prepared using original data by the authors. Thus, the maps for the Moscow Region were built on the formal territorial classification with reference of the information to a degree-grid (5 × 5 km). This method was used for other regions to represent disease incidence, occurrence of pathogens, and distribution of potentially dangerous sites, such as anthrax animal burial sites. The authors also compiled regional maps on the nosological ranges of HFRS, leptospirosis and tularemia.

The primary scales of the maps for the territory of Russia are 1:20,000,000 and 1:30,000,000, and 1:4,000,000 and 1:10,000,000 for the maps of individual regions.

## Results and discussion

To illustrate the opportunities of the atlas let us consider ixodid tick-borne borrelosis as one of the most important natural-focal diseases in Russia. A considerable part of world’s areal of this disease lies within the Russain territory. Accounting for the number of cases tick-borne borrelosis constitutes one of the major natural-focal infections.

A series of maps of disease morbidity that was developed allows us to comprehensively analyze of the epidemiological situation.The inventory map of mean annual morbidity encompasses 73 administrative units where this disease was registered (Figure 1). Autochtonous cases were not registered in nine, mostly southern, administrative units: Northern Caucasus, Saratov, Astrakhan and Rostov regions, as well as in one north-eastern unit, namely Chukotka district. The map shows that morbidity varies from 0,02 (Krasnodar krai) to 42,0 (Tomsk oblast) per 100 000 population.

In general, from 1997 to 2010 the morbidity index has an undulating character with minimums in 1997 and 2004 (approximately 4,5 cases per 100 000 population) and peaks in 2003 and 2009 (upward of 6,0 cases per 100 000 population).

The map of types of dynamics of tick-borne borreliosis which was created on the basis of an original method of tipology classification, allows us to combine all regions into five major categories. The most regions are of the type with a low morbidity level (less than 1,0 per 100.000) and with a steady mode with virtually constant levels of annual morbidity. The worst situation is associated with latitudes between 52° and 61° N (Udmurt republic, Kostroma, Yaroslavl, Vologda, Kirov and Tomsk regions), where the foci are connected with landscapes of temperate broadleaf and mixed forests and southern taiga. Those regions are marked with a high level of morbidity with distinctive peaks in several year.The map of disease dynamics, made as ring cartogram, further defines the dynamics of morbidity for each region per year (Figure 2).The role of tick-borne borreliosis, or of any other infection, in the overall natural-focal disease morbidity can be assessed with the help of the “Nosological profiles” map. (Figure 3). Along with that, the “Nosological Profiles” map presents the characteristics of the distribution of the most important natural-focal diseases at the national level and can be used to assess the representativeness of the nosological units and frequency of the manifestation of certain infections in different regions. It reflects the most common natural-focal infections in Russia, among which, as the map’s analysis has shown, the leading role is played by tick-borne borreliosis, leptospirosis, and HFRS, which occur in most parts of Russia, as well as by tick-borne encephalitis and opisthorchiasis. The map is also remarkable because it reflects the annual changes of the range of natural-focal diseases observed on the Russian territory.

Mapping the morbidity for such large territorial units as the federal administrative units of the Russian Federation is a necessary but not sufficient element in the assessment of natural-focal disease spread. This representation is an epidemiological characterization of the population rather than that of specific diseases, whose ranges are determined primarily by the parameters of the environment. The nature of the information on disease incidence in the population at a small scale (i.e., covering a large area) does not allow for use of the natural boundaries. In order to overcome this limitation, the maps at the federal level are supplemented with regional and more detailed maps that contain an in depth assessment of the character of distribution of the foci and spread of the natural-focal diseases in the federal administrative units of the Russian Federation.

The maps created for key regions, based on case localization, allow for a detailed look at the situation. For example, local foci of leptospirosis and tularemia have been discovered in Moscow region.

## Conclusions

The Atlas of Russia “Natural-Focal Diseases” sums up a large volume of information on natural-focal diseases in the Russian Federation as a whole; it is assembled in a format open to the public. It can be used to assess the level of knowledge on some natural-focal diseases in Russia and to identify the regions that require additional targeted research efforts.

The compiled series of maps in the Atlas allows for:

• a systematization and analysis of the role of natural and socioeconomic factors in the spread of natural-focal infections;

• an identification of the spectrum of the most diagnosed natural-focal diseases observed over the past 15years at the level of the administrative units of the Russian Federation and the country as a whole;

• an identification of the most active foci and assessment of their potential danger to humans;

• a quantification of disease morbidity in both absolute and relative terms in general and in some model regions;

• a forecast of disease incidence based on the types of dynamics of disease incidence using mathematical-cartographic modeling for the current natural-focal diseases;

• an identification the most visual ways of cartographic representation of the dynamics of disease incidence;

• carrying out medico-geographical analysis of the territory of the spread of the basic nosoforms of natural-focal diseases in the regions of the Russian Federation and in the territory of Russia as a whole.

Taken together, the maps allow for an assessment of the persistence in disease manifestation and the degree of specific disease spread risk in the territories. The results of analysis can be used for health monitoring and for the development of targeted preventive measures, especially in areas of new economic expansion and areas affected by the recreational load.

The ultimate goal of the Atlas is to promote an objective assessment of the medico-geographical situation in relation to a complex of natural-focal diseases, its control, and science-based response activities of health authorities in emergency situations in Russia and outside its national borders.

## Competing interests

The authors declare that they have no competing interests.

## Authors’ contributions

All authors conceived of the study and participated in the design. All authors contributed to the writing of, reviewed and approved the final manuscript.
